# Diagnostic Efficiency of Pan-Immune-Inflammation Value to Predict Prostate Cancer in Patients with Prostate-Specific Antigen between 4 and 20 ng/mL

**DOI:** 10.3390/jcm12030820

**Published:** 2023-01-19

**Authors:** Meikai Zhu, Yongheng Zhou, Zhifeng Liu, Zhiwen Jiang, Wenqiang Qi, Shouzhen Chen, Wenfu Wang, Benkang Shi, Yaofeng Zhu

**Affiliations:** 1 Department of Urology, Qilu Hospital of Shandong University, Jinan 250012, China; 2 Department of Urology, Tai’an City Central Hospital, Tai’an 271000, China

**Keywords:** PIV, systemic inflammatory markers, prostate biopsy, diagnosis, prostate cancer

## Abstract

Introduction: To evaluate the predictive value of the pan-immune-inflammation value (PIV) and other systemic inflammatory markers, including the neutrophil-to-lymphocyte ratio (NLR), derived neutrophil-to-lymphocyte ratio (dNLR), monocyte-to-lymphocyte ratio (MLR), platelet-to-lymphocyte ratio (PLR), and systemic immune-inflammation index (SII), for prostate cancer (PCa) and clinically significant prostate cancer (CSPCa) in patients with a prostate-specific antigen (PSA) value between 4 and 20 ng/mL. Patients and Methods: The clinical data of 319 eligible patients who underwent prostate biopsies in our hospital from August 2019 to June 2022 were retrospectively analyzed. CSPCa was defined as a “Gleason grade group of ≥2”. A univariable logistic regression analysis and multivariable logistic regression analysis were conducted to analyze the association between the PIV, SII, MLR, and PCa/CSPCa. For the inflammatory indicators included in the multivariable logistic regression analysis, we constructed models by combining the separate inflammatory indicator and other significant predictors and compared the area under the curve (AUC). A nomogram based on the PIV for PCa was developed. Results: We included 148 PCa patients (including 127 CSPCa patients) and 171 non-PCa patients in total. The patients with PCa were older, had higher MLR, SII, PIV, and total PSA (TPSA) values, consumed more alcohol, and had lower free/total PSA (f/T) values than the other patients. Compared with the non-CSPCa group, the CSPCa group had higher BMI, MLR, PIV, TPSA values, consumed more alcohol, and had lower f/T values. The univariable regression analysis showed that drinking history, higher MLR, PIV, and TPSA values, and lower f/T values were independent predictors of PCa and CSPCa. The AUC of the PIV in the multivariable logistic regression model was higher than those of the MLR and SII. In addition, the diagnostic value of the PIV + PSA for PCa was better than the PSA value. However, the diagnostic value for CSPCa was not significantly different from that of using PSA alone, while the AUC of the PIV + PSA was higher than the individual indicator of the PSA value. Conclusions: Our study suggests that for the patients who were diagnosed with PSA values between 4 and 20 ng/mL, the PIV and MLR are potential indicators for predicting PCa and CSPCa. In addition, our study indicates that the new inflammatory index PIV has clinical value in the diagnosis of PCa and CSPCa.

## 1. Introduction

Prostate cancer (PCa) is a common malignant tumor worldwide and is the second cause of cancer-related death in men [1]. Prostate-specific antigen (PSA) is a major biomarker for PCa diagnosis. Currently, PCa is usually determined by systematic ultrasound-guided biopsies prompted by elevated levels of PSA in serum [2]. However, only 25% of men with elevated PSA levels are diagnosed with PCa because of the poor specificity of PSA, which means that 75% of patients undergo unnecessary and potentially harmful follow-up tests, such as biopsies, especially for men with PSA values between 4.0 and 20.0 ng/mL (low and medium clinical risk category) [3]. In order to make up for this defect, many biomarkers of PCa have been developed successively, such as the prostate health index, 4K score, SelectMDx, and ExoDx Prostate IntelliScoreTM [4]. However, these experimental methods have some disadvantages, such as high costs, which means they cannot be routinely used for the detection of PCa [5].

In recent years, it has become increasingly accepted that certain systemic inflammatory reactions may play a significant role in tumor promotion and progression. Tumor-related systemic inflammatory markers, including the neutrophil-to-lymphocyte ratio (NLR), derived neutrophil-to-lymphocyte ratio (dNLR), monocyte-to-lymphocyte ratio (MLR), platelet-to-lymphocyte ratio (PLR), and systemic immune-inflammation index (SII), have gained attention as diagnostic tools for tumors [6,7].

The pan-immune-inflammation value (PIV), a novel equation that includes the neutrophil count, platelet count, monocyte count, and lymphocyte count from peripheral blood, has been reported as a potential prognostic biomarker in several cancers [8]. There have been no studies of the diagnostic value of the PIV in PCa.

In the present study, our primary goal was to investigate whether the PIV could be used to predict PCa in patients with PSA levels between 4.0 and 20.0 ng/mL. We also verified the diagnostic efficacy of the NLR, dNLR, MLR, PLR and SII in PCa.

## 2. Materials and Methods

### 2.1. Patient Selection Information Collection

This is a retrospective study that was approved by the Institutional Ethics Review Board of QILU Hospital of Shandong University (KYLL-202111-107). We obtained the information of all patients who received prostate biopsies with PSA levels of 4.0–20.0 ng/mL in our hospital from August 2019 to June 2022 from the electronic medical record system at our hospital. All patients underwent routine blood tests with serum PSA derivative (including total PSA [TPSA] and free PSA [fPSA]) within 2 weeks before their biopsies. Patients with one or more of the following conditions were excluded from this study: (I) Patients with other malignancies, known infections, and hematological diseases; (II) Patients who had had prostate surgery (such as transurethral resection of the prostate) before their biopsies; (III) Patients with pathological diagnoses of atypical small acinar proliferation and prostatic intraepithelial neoplasia; and (IV) Patients with incomplete clinical data. Then, we collected the following data of eligible patients from the medical records: age, body mass index (BMI), history of tobacco and alcohol use, medical history, blood test results with serum PSA, histopathologic findings, and Gleason score.

### 2.2. Biopsy Method and Pathological Examination

All patients had received prostate mpMRI before biopsies, which were performed by two uroradiologists with a minimum of three years of experience using a 3.0 T scanner. Experienced members of the surgical team retrospectively performed imaging assessments to reach a consensus on the imaging findings to determine the biopsy methods. Finally, all patients underwent transrectal biopsies or transperineal biopsies under local anesthesia. The prostate biopsies were performed with 12 + 3 cores (on the basis of 12 systematic cores, with the remaining core at the suspicious area shown on the MRI by cognitive fusion biopsies). Then, pathological tissues from the biopsy specimens were analyzed by two experienced uropathologists according to International Society of Urological Pathology consensus guidelines within one week post-surgery.

### 2.3. Data Management

The patients were classified into non-PCa group and PCa group based on the histopathologic results. In addition, we divided the patients into CSPCa group and non-CSPCa group. The definition of clinically significant prostate cancer (CSPCa) was “Gleason grade group of ≥2” [9]. The PIV, NLR, dNLR, MLR, PLR and SII were defined as “neutrophil count × platelet count × monocyte count/lymphocyte count”, “neutrophil count/lymphocyte count”, “neutrophil count/(leukocyte count–neutrophil count)”, “monocyte count/lymphocyte count”, “platelet count/lymphocyte count” and “neutrophil count × platelet count/lymphocyte count”, respectively. All the above blood cell counts were obtained within two weeks before biopsies.

### 2.4. Statistical Analysis

All continuous variables were tested for normality. The continuous variables that met the normality test used the Student’s *t*-test and the variables with skewed distribution used the Mann–Whitney *U*-test. Continuous variables with normal distribution were reported with mean ± SD and continuous variables with skewed distribution were reported as median (IQR). Categorical variables were analyzed using Chi-square tests and reported as numbers (percentages). Univariable and multivariable logistic regression analyses were conducted to identify the independently predictive factors for PCa and CSPCa. The predictors with *p* values less than 0.05 in univariable logistic regression were included in multivariable logistic regression. We constructed different models using different inflammatory factors and other clinical variables and compared the performance of different models. A *p* value less than 0.05 was considered statistically significant. We developed a PCa risk nomogram, including PIV for prostate biopsy. The calibration was examined by the calibration curves. Decision curve analysis (DCA) was performed to assess the clinical usefulness of the nomogram by calculating the net benefits. The DeLong test was used to compare the differences in AUC. SPSS V.25.0 (IBM Corp, Armonk, NY, USA) and R statistical software (Version 4.1.0) were used to perform statistical analysis.

## 3. Results

### 3.1. Clinical Demographics of the Eligible Patients

A total of 319 individual patients met the study’s entry criteria and were included in the study. The mean age, PIV, TPSA levels were 66 years, 197.04, and 9.23ng/mL, respectively. PCa was detected in 148 patients (including 127 patients with CSPCa). The characteristics and laboratory values of the patients are shown in Table 1.

The mean age (67.00 vs 65.00, *p* = 0.011), MLR (0.30 vs 0.25, *p* < 0.001), SII (427.26 vs 393.89, *p* = 0.03), PIV (229.62 vs 171.54, *p* < 0.001), and TPSA (10.76 vs 8.40, *p* < 0.001) of the PCa group were significantly higher than those of the non-PCa group. In addition, the proportion of patients with a history of alcohol use in the PCa group was also higher than that in the non-PCa group (Table 1).

The CSPCa group had higher BMI, MLR, PIV and TPSA levels than the non-CSPCa group. However, there was no significant difference in the age or SII between the two groups. Moreover, there was no statistically significant difference in the NLR, dNLR, and PLR between the PCa group vs non-PCa group as well as the CSPCa group vs non-CSPCa group (Table 1).

### 3.2. Univariable and Multivariable Analyses of Clinical Indicators

We conducted univariable and multivariable logistic regression analyses to determine the predictive factors of the clinical indicators. Age, history of alcohol use, MLR, SII, PIV, TPSA and f/T values were significant predictors of PCa according to the results of the univariable regression analysis (Table 2). Then we chose all the significant variables in the univariable regression analysis and subjected them to the multivariable regression analysis. The results showed that history of alcohol use, higher age, higher MLR and TPSA values, and lower f/T values had a greater probability for the detection of PCa (Table 2).

In the univariable regression analysis between the CSPCa group and non-CSPCa group, we found that patients with higher BMIs, a greater history of alcohol use, higher MLR, PIV, and TPSA values, and lower f/T values were more likely to be diagnosed with CSPCa (Table 2). The independent variables with *p* < 0.05 in the univariable analysis were selected for the multivariable regression analysis. According to the result of the multivariable logistic regression analysis, higher BMI, MLR, and TPSA values and lower f/T values were the independent predictors of CSPCa (Table 2).

### 3.3. Multivariable Logistic Regression Analysis of Different Models of Inflammatory Markers

**Figure 1:**
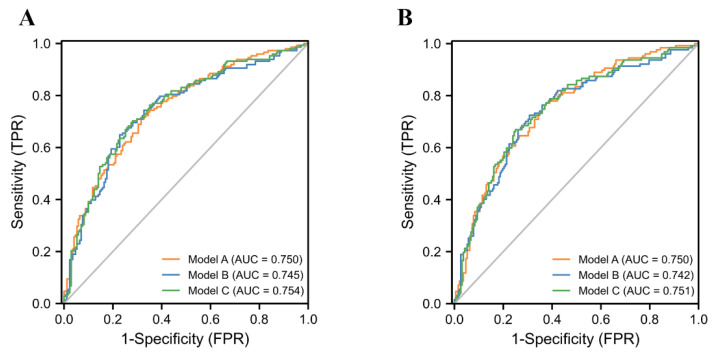
The AUC curves of different models based on SII, MLR, and PIV, respectively, in prostate cancer (**A**) and clinically significant prostate cancer (**B**).

As mentioned above, we found that age, BMI, history of alchohol use, TPSA, f/T values were independent predictors of PCa or CSPCa. Therefore, we performed multivariable logistic regression analyses with MLR, SII, PIV, and other risk factors and constructed different models (Model A, Model B, Model C) (Table 3), respectively, to predict the outcomes of the biopsies. The AUC values (Figure 1) from high to low were Model C (AUC = 0.754, 95% CI: 0.701–0.808, *p* = 0.001), Model A (AUC = 0.750, 95% CI: 0.701–0.808, *p* < 0.001), and Model B (AUC = 0.745, 95% CI: 0.701–0.808, *p* = 0.008). This meant that among the three models, Model C had the highest diagnostic value for the detection of PCa, although the difference between them is not obvious. The specificity and sensitivity of Model C for the PCa values were 0.703 and 0.719 (Table 4), respectively. Similar results appeared in the group of CSPCa patients, which showed that Model C (AUC = 0.751, 95% CI: 0.696–0.806, *p* = 0.003) had the highest diagnostic value followed by Model A (AUC = 0.750, 95% CI: 0.696–0.804, *p* = 0.001) and Model B (AUC = 0.742, 95% CI: 0.686–0.798, *p* = 0.021) with a sensitivity and specificity of 0.669 and 0.750 (Table 4).

### 3.4. ROC Curve Analysis of Variables

**Figure 2:**
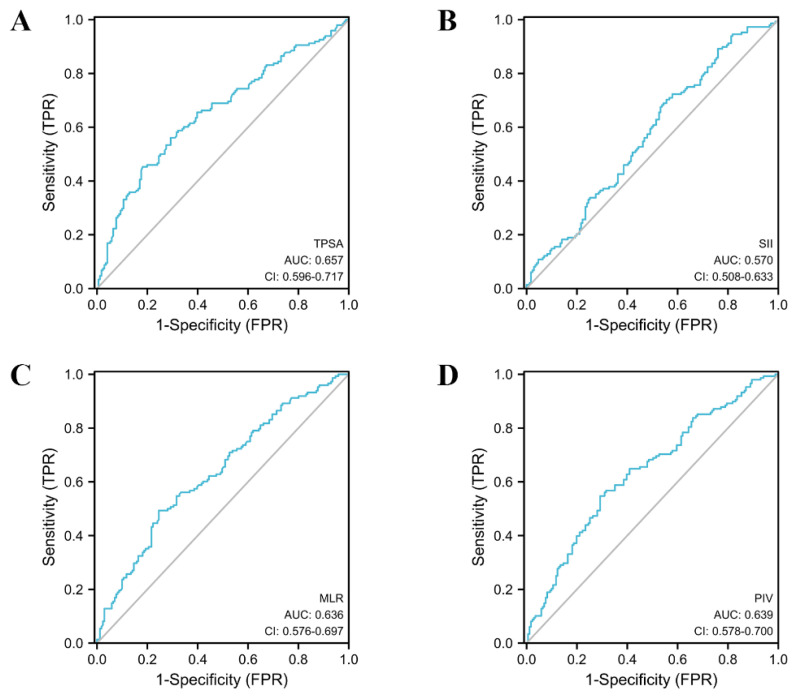
The AUC curves of TPSA and inflammatory markers in prostate cancer. (**A**): The AUC curves of TPSA; (**B**): The AUC curves of SII; (**C**): The AUC curves of MLR; (**D**): The AUC curves of PIV.

**Figure 3:**
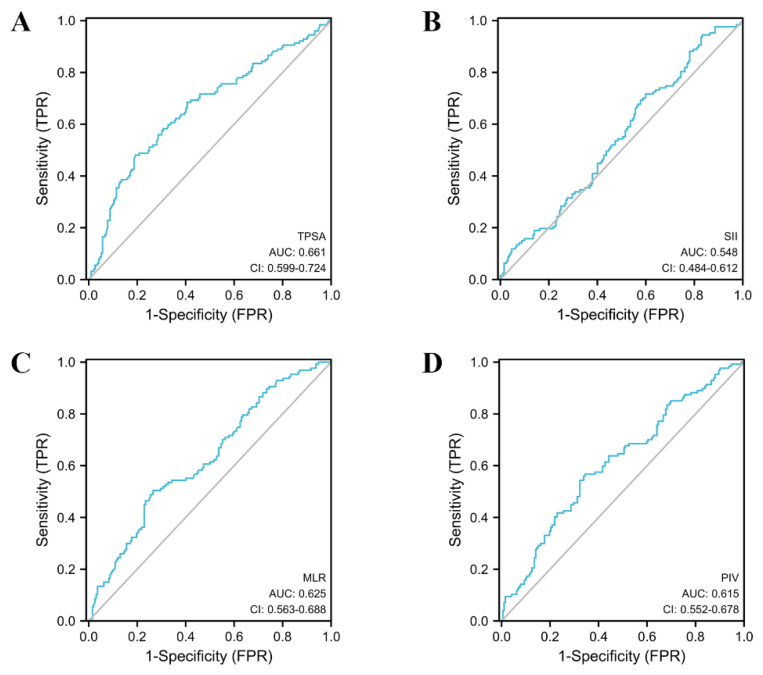
The AUC curves of TPSA and inflammatory markers in clinically significant prostate cancer. (**A**): The AUC curves of TPSA; (**B**): The AUC curves of SII; (**C**): The AUC curves of MLR; (**D**): The AUC curves of PIV.

In order to evaluate the diagnostic value of a single variable for PCa and CSPCa, we performed the ROC-AUC analysis of the MLR, SII, PIV and TPSA. The detailed results of the analysis are presented in Table 4, Figure 2 and Figure 3. TPSA (AUC = 0.657, 95% CI: 0.596–0.717) had the highest predictive value for PCa values according to the parameters of the analysis. The AUC values for the MLR, SII, and PIV were 0.636 (95% CI: 0.576–0.697), 0.570 (95% CI: 0.508–0.633), and 0.639 (95% CI: 0.578–0.700), respectively. In the group of CSPCa, the ROC curve analysis showed that the AUCs of MLR, SII, PIV, and TPSA were 0.625 (95% CI: 0.563–0.688), 0.548 (95% CI: 0.484–0.612), 0.615 (95% CI: 0.552–0.678), and 0.661 (95% CI: 0.599–0.724), respectively. In summary, TPSA has the highest diagnostic value for both PCA and CSPCa. At the same time, the PIV and MLR are also the powerful predictors, although not as good as TPSA. The predictive value of the SII for PCA and CSPCa is not excellent.

In addition, we also used the Delong test to compare the diagnostic efficacy of TPSA +PIV compared with using TPSA alone for PCa/CSPCa. The diagnostic efficacy of TPSA + PIV (AUC = 0.700, 95% CI: 0.642–0.757) for PCa is higher than that of TPSA (AUC = 0.657, 95% CI: 0.596–0.717) with a statistical difference (*p* = 0.02).

### 3.5. Development of a Nomogram for PCa Prediction

**Figure 4:**
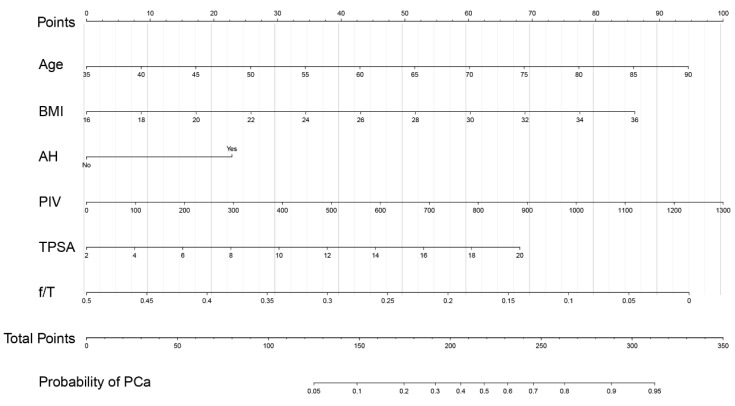
Nomogram for predicting PCa based on the training cohort. The prostate biopsy nomogram was developed in the training cohort, with age, BMI, AH, PIV, TPSA, and f/T incorporated. BMI: body mass index; AH: alcohol history; PIV: pan-immune-inflammation value; TPSA: total prostatic specific antigen; f/T: free/total prostatic specific antigen ratio.

In order to intuitively show the predictive value of the PIV for PCa, we developed a nomogram (Figure 4) for positive biopsy prediction in prostate biopsy patients based on Model C; patients in this study were randomly divided into a training group and validation group according to the random number table in a 3:1 ratio. The calibration curve of the nomogram demonstrated good agreement between prediction and observation in the training group (Supplementary Figure S1A) and validation group (Supplementary Figure S1B). The decision curve analysis (Supplementary Figure S2) illustrated that the nomogram model has excellent clinical application.

## 4. Discussion

In this retrospective study of patients undergoing prostate biopsies with PSA values of 4.0–20.0ng/mL, we found that compared with non-PCa patients, the PIV and MLR of the patients with PCa were significantly higher. In addition, the PIV and MLR showed high predictive values in both the univariable prediction models and the multivariable prediction models of PCa and CSPCa. The SII was also significantly elevated in the PCa patients, but there was no significant difference between the CSPCa and Non-CSPCa groups, and its predictive value for PCa and CSPCa was not as good as the MLR and PIV. Meanwhile, other systemic inflammatory markers, such as the NLR, dNLR, and PLR, had limited diagnostic value for PCa and CSPCa.

Inflammation within the tumor microenvironment has effects that promote malignant transformations in cells, as well as carcinogenesis and its progression [10]. Inflammation not only works as a promoter during carcinogenesis (inflammation-induced cancer), but growing tumors that escape immunosurveillance also induce an inflammatory response that can support cancer progression (cancer-related inflammation) [11]. More and more studies show that with the occurrence and progression of cancer, a series of changes will occur in inflammatory-related cells and inflammatory-related substances in patients, which are closely related to the diagnosis and prognosis of tumors, such as the systemic increase in neutrophils [12], elevated levels of circulating monocytes [13], thrombocytosis [14], and lymphopenia [15]. The PIV incorporates the above inflammatory indicators into one equation, and several studies have confirmed that the PIV has a good predictive effect on the prognosis of some tumor patients. Our study explored the diagnostic value of the PIV in PCa for the first time and proved that the PIV is a significant predictor for PCa and CSPCa [8].

A growing number of studies have explored the potential diagnostic value of different kinds of systemic inflammatory indicators in PCa. However, contradictory results were reported from these studies. A retrospective study by Pawel et al. [16] found that the MLR was not helpful for the diagnosis of PCa. Another four studies, including a large retrospective analysis [17,18,19,20], showed that the MLR is an important predictor in PCa diagnosis, which is consistent with our findings. In conclusion, we believe that a higher MLR level is significantly related to the detection of PCa/CSPCa. The diagnostic value of the NLR, PLR, and SII for PCa has been controversial. The research of Durvesh [21] and Hiroshi [22] showed that the NLR is a good predictor of PCa, while the research of Du [23] showed that the NLR has a limited diagnostic value for PCa. The conclusions of other studies [16,18,19] are the same as the outcomes of Du’s study. Our study showed that the NLR may not be a valuable predictor of PCA/CSPCa. The results of two studies [24,25] pointed out that a higher PLR is related to the detection of PCa, while other studies [16,18,19,26,27] showed that there is no obvious relationship between the PLR and PCa. A similar situation has been found in studies of the SII [16,24,27]. In our study, the PLR has no significance in the diagnosis of PCa; although the SII had a certain diagnostic value for PCa, it is not as good as the PIV and MLR. We believe that the reasons for the above contradictory conclusions may be mainly related to the inconsistent clinical risk stratification of the patients participating in the study. Some studies included low-risk patients with PSA values of 4–10 ng/mL before their biopsies, which means that the changes in the systemic inflammation indicators in these patients may not be obvious, and patients were not grouped according to PSA levels in other studies. In addition, the date of blood sample collection before the biopsies and the method of the biopsies also have a certain impact on the results of the study.

To our knowledge, this study is the first report on the predictive effect of the PIV on PCa. In our study, we compared the superiority of the PIV with that of other systemic inflammatory indices, such as the MLR and SII. Compared with PIV, MLR and SII did not include the neutrophil count × platelet count and monocyte count, respectively. The PIV showed good diagnostic value, both for PCa and CSPCa. After a comprehensive consideration of the patients’ age, BMI, history of alcohol use, and TPSA and f/T values, the diagnostic value of the PIV (model C) for PCA/CSPCa is better than that of the MLR (model A) and the SII (model B). The ROC analysis and the result of the prognostic model showed that the PIV was better than the MLR and SII in its comprehensive value regarding the prediction of PCa. It should be noted that the interaction among inflammation, immunity, and cancers is complex and interlocking. The PIV was created to involve more mediators in the immune-inflammatory markers to more accurately model and reflect the inflammatory environment in patients with PCa so that it has better predictive power than incomplete systemic inflammatory indices. In addition, in the comparison of the diagnostic value of TPSA and TPSA + PIV for PCa, the combination of PIV + TPSA (AUC = 0.700, 95CI: 0.642–0.757) was proven to be more significant than TPSA (AUC = 0.657, 95CI: 0.596–0.717) (*p* = 0.02). These findings of this study may have significant clinical implications. This is because, although PSA screening has been widely implemented in decision making for prostate biopsies in clinical practice [28], its low specificity for PCa generally leads to many unnecessary biopsies [29]. According to the results of our study, the PIV, which can be measured easily, was shown to be a feasible index that can be used in any clinical setting. Most importantly, the combination of the PIV and TPSA likely improves the accuracy of prostate biopsies in patients with PSA values of 4.0–20.0 ng/mL, and it may be useful for making better preoperative assessments and individualized treatment decisions. These findings suggest that it is necessary to perform the routine blood tests as a routine test in such patients. In the current study, we chose the PIV, together with age, BMI, history of alcohol use, and TPSA and f/T values to develop a new nomogram to predict PCa risk. Our nomogram had a good potential for discrimination and calibration, which was confirmed by the internal validation. Unfortunately, we did not conduct an independent external validation due to the research conditions.

In addition, we found that higher age, BMI, and alcohol consumption were directly related to the diagnosis of PCa, which was consistent with previous reported results [30,31,32]. This knowledge could contribute to more efficient risk factor management in populations, which can aid in the prevention of PCa, significantly reducing the impact of this disease on public health.

This study is a single-institution retrospective analysis and therefore has some inherent limitations. Moreover, we did not distinguish between the biopsy strategies because of the limited sample size. Large-scale, multicenter studies are warranted to confirm our findings in the future.

## 5. Conclusions

Our study shows that the PIV and MLR are significant predictors of PCa and CSPCa diagnoses in patients with PSA levels from 4.0 to 20.0 ng/mL, and they may be useful to avoid unnecessary biopsies or biopsy-related morbidities in real clinical practice. The NLR, dNLR, PLR and SII may have a limited role in predicting PCa or CSPCa.

## Figures and Tables

**Table 1:** Characteristic baseline.

Variable	Overall (*n* = 319)	Non-PCa (*n* = 171)	PCa (*n* = 148)	*p* Value	Non-CSPCa (*n* = 192)	CSPCa (*n* = 127)	*p* Value
Age, year	66.00 (61.00–72.00)	65.00 (59.00–71.00)	67.00 (62.00–73.00)	0.011	66 (60–72)	66 (62–73)	0.285
BMI, kg/m^2^	24.80 (22.99–26.57)	24.57 (22.78–26.12)	25.02 (23.54–27.03)	0.064	24.57 (22.78–26.12)	25.10 (23.56–27.06)	0.025
SH (%)				0.191			0.189
Y	90 (28.2)	43 (25.1)	47 (31.8)		49 (25.5)	41 (32.3)	
N	229 (71.8)	128 (74.9)	101 (68.2)		143 (74.5)	86 (67.7)	
AH (%)				0.015			0.016
Y	87 (27.3)	37 (21.6)	50 (33.8)		43 (22.4)	44 (34.6)	
N	232 (72.7)	134 (78.4)	98 (66.2)		149 (77.6)	83 (65.4)	
NLR	1.90 (1.52–2.48)	1.88 (1.44–2.58)	1.93 (1.59–2.47)	0.171	1.89 (1.44–2.51)	1.93 (1.58–2.48)	0.244
dNLR	1.37 (1.10–1.76)	1.40 (1.09–1.86)	1.36 (1.12–1.72)	0.679	1.40 (1.09–1.78)	1.35 (1.11–1.73)	0.720
MLR	0.27 (0.22–0.34)	0.25 (0.20–0.30)	0.30 (0.23–0.39)	<0.001	0.26 (0.20–0.31)	0.30 (0.23–0.39)	<0.001
PLR	122.98 (98.58–150.50)	120 (96.11–143.86)	132.30 (101.16–153.20)	0.053	121.10 (96.34–144.56)	132.54 (100.00–153.21)	0.099
SII, 10^9^	411.79 (316.84–531.05)	393.89 (293.30–501.92)	427.26 (339.28–544.45)	0.030	402.14 (305.01–521.53)	423.77 (328.05–531.39)	0.145
PIV, 10^18^	197.04 (134.56–289.76)	171.54 (123.48–244.10)	229.62 (152.47–329.29)	<0.001	181.04 (125.45–251.83)	228.49 (151.17–325.61)	0.001
Hb, g/L	147 (138–154)	148 (139–155)	147 (137–154)	0.448	148 (139–155)	147 (137–154)	0.484
TPSA, ng/mL	9.23 (6.71–12.53)	8.40 (6.07–10.93)	10.76 (7.65–14.27)	<0.001	8.40 (6.10–11.07)	11.05 (8.01–14.52)	<0.001
fPSA, ng/mL	1.20 (0.82–1.74)	1.22 (0.86–1.76)	1.14 (0.79–1.66)	0.792	1.23 (0.85–1.78)	1.14 (0.80–1.60)	0.609
f/T	0.14 (0.10–0.18)	0.15 (0.11–0.19)	0.12 (0.79–1.66)	<0.001	0.15 (0.11–0.19)	0.11 (0.09–0.16)	<0.001

BMI: body mass index; SH: smoking history; AH: alcohol history; NLR: neutrophil-to-lymphocyte ratio; dNLR: derived neutrophil-to-lymphocyte ratio; MLR: monocyte-to-lymphocyte ratio; PLR: platelet-to-lymphocyte ratio; SII: systemic immune-inflammation index; PIV: pan-immune-inflammation value; HB: hemoglobin; TPSA: total prostatic specific antigen; fPSA: free prostatic specific antigen; f/T: free/ total prostatic specific antigen ratio; PCa: prostate cancer; CSPCa: clinically significant prostate cancer, which was defined as Gleason grade ≥ 2.

**Table 2:** Univariable and multivariable analyses of clinical indicators.

PCa	Univariable Regression Analysis		Multivariable Regression Analysis		CSPCa	Univariable Regression Analysis		Multivariable Regression Analysis	
	OR (95% CI)	*p* Value	OR (95% CI)	*p* Value		OR (95% CI)	*p* Value	OR (95% CI)	*p* Value
Age	1.035 (1.008–1.064)	0.012	1.046 (1.014–1.079)	0.005	Age	1.015 (0.988–1.042)	0.284		
BMI	1.075 (0.995–1.160)	0.066			BMI	1.090 (1.007–1.179)	0.032	1.114 (1.021–1.217)	0.016
SH					SH				
Y	1.385 (0.849–2.259)	0.192			Y	1.391 (0.849–2.279)	0.190		
N	1				N	1			
AH					AH				
Y	1.848 (1.122–3.042)	0.016	1.975 (1.141–3.416)	0.015	Y	1.837 (1.116–3.025)	0.017	1.706 (0.989–2.940)	0.055
N	1		1		N	1		1	
NLR	1.234 (0.936–1.629)	0.136			NLR	1.192 (0.903–1.574)	0.216		
dNLR	0.849 (0.636–1.134)	0.268			dNLR	0.862 (0.642–1.158)	0.324		
MLR	52.028 (7.377–366.922)	<0.001	16.513 (1.091–249.847)	0.043	MLR	27.469 (4.298–175.552)	<0.001	19.473 (1.557–243.616)	0.021
PLR	1.003 (0.998–1.008)	0.188			PLR	1.003 (0.998–1.007)	0.299		
SII	1.001 (1.000–1.002)	0.028	1.000 (0.998–1.002)	0.731	SII	1.001 (1.000–1.002)	0.053		
PIV	1.003 (1.001–1.004)	0.001	1.002 (0.998–1.005)	0.324	PIV	1.002 (1.001–1.003)	0.002	1.001 (0.999–1.003)	0.406
Hb	0.997 (0.983–1.011)	0.684			Hb	0.998 (0.984–1.013)	0.814		
TPSA	1.163 (1.094–1.236)	<0.001	1.138 (1.063–1.217)	<0.001	TPSA	1.158 (1.090–1.231)	<0.001	1.140 (1.067–1.218)	<0.001
fPSA	1.076 (0.794–1.459)	0.635			fPSA	0.941 (0.689–1.286)	0.704		
f/T	0.003 (0.000–0.096)	0.001	0.006 (0.000–0.413)	0.018	f/T	0.001 (0.000–0.028)	<0.001	0.004 (0.000–0.278)	0.010

BMI: body mass index; SH: smoking history; AH: alcohol history; NLR: neutrophil-to-lymphocyte ratio; dNLR: derived neutrophil-to-lymphocyte ratio; MLR: monocyte-to-lymphocyte ratio; PLR: platelet-to-lymphocyte ratio; SII: systemic immune-inflammation index; PIV: pan-immune-inflammation value; HB: hemoglobin; TPSA: total prostatic specific antigen; fPSA: free prostatic specific antigen; f/T: free/total prostatic specific antigen ratio; PCa: prostate cancer; CSPCa: clinically significant prostate cancer, which was defined as Gleason grade ≥ 2; OR: odds ratio; CI: CI: confidence interval.

**Table 3:** Multivariable logistic regression analysis of different models of inflammatory markers.

PCa	Model A		Model B		Model C	
	OR (95% CI)	*p* Value	OR (95% CI)	*p* Value	OR (95% CI)	*p* Value
Age	1.048 (1.016–1.081)	0.003	1.056 (1.023–1.089)	0.001	1.055 (1.023–1.089)	0.001
BMI	1.106 (1.014–1.207)	0.023	1.111 (1.019–1.212)	0.017	1.110 (1.017–1.211)	0.019
AH		0.031		0.029		0.032
Y	1.841 (1.059–3.200)		1.841 (1.063–3.188)		1.832 (1.054–3.186)	
N	1		1		1	
MLR	59.057 (7.385–472.306)	<0.001	/		/	
SII	/		1.001 (1.000–1.003)	0.008	/	
PIV	/		/		1.003 (1.001–1.004)	0.001
TPSA	1.143 (1.068–1.223)	<0.001	1.138 (1.064–1.217)	<0.001	1.134 (1.061–1.213)	<0.001
f/T	0.004 (0.000–0.260)	0.009	0.003 (0.000–0.215)	0.007	0.004 (0.000–0.238)	0.008
AUC (95% CI)	0.750 (0.697–0.804)		0.745 (0.701–0.808)		0.754 (0.701–0.808)	
**CSPCa**						
Age	1.025 (0.994–1.058)	0.111	1.033 (1.002–1.065)	0.040	1.032 (1.001–1.064)	0.045
BMI	1.119 (1.024–1.222)	0.013	1.123 (1.028–1.226)	0.010	1.122 (1.027–1.226)	0.011
AH		0.049		0.050		0.055
Y	1.732 (1.002–2.996)		1.721 (1.000–1.226)		1.706 (0.988–2.945)	
N	1		1		1	
MLR	34.010 (4.624–250.170)	0.001	/		/	
SII	/		1.001 (1.000–1.002)	0.021	/	
PIV	/		/		1.002 (1.001–1.004)	0.003
TPSA	1.135 (1.062–1.213)	<0.001	1.131 (1.059–1.208)	<0.001	1.126 (1.054–1.203)	<0.001
f/T	0.002 (0.000–0.122)	0.004	0.001 (0.000–0.100)	0.003	0.001 (0.000–0.104)	0.003
AUC (95% CI)	0.750 (0.696–0.804)		0.742 (0.686–0.798)		0.751 (0.696–0.806)	

AH: alcohol history; MLR: monocyte-to-lymphocyte ratio; SII: systemic immune-inflammation index; PIV: pan-immune-inflammation value; TPSA: total prostatic specific antigen; f/T: free/total prostatic specific antigen ratio; AUC: the area under curve; CI: Confidence interval; OR: odds ratio; PCa: prostate cancer; CSPCa: clinically significant prostate cancer, which was defined as Gleason grade ≥ 2.

**Table 4:** ROC curve analysis of variables.

Variables	AUC (95% CI)	Cut-Off	Sensitivity	Specificity	PPV	NPV	Youden Index
**PCa**							
MLR	0.636 (0.576–0.697)	0.302	0.493	0.754	0.635	0.632	0.248
SII	0.570 (0.508–0.633)	374.674	0.703	0.444	0.523	0.633	0.147
PIV	0.639 (0.578–0.700)	219.616	0.547	0.708	0.618	0.644	0.255
TPSA	0.657 (0.596–0.717)	11.577	0.453	0.819	0.684	0.633	0.271
Model A	0.750 (0.697–0.804)	0.382	0.709	0.684	0.660	0.731	0.394
Model B	0.745 (0.690–0.800)	0.425	0.649	0.772	0.711	0.717	0.421
Model C	0.754 (0.701–0.808)	0.402	0.703	0.719	0.684	0.737	0.422
**CSPCa**							
MLR	0.625 (0.563–0.688)	0.302	0.504	0.734	0.557	0.691	0.238
SII	0.548 (0.484–0.612)	365.367	0.717	0.401	0.442	0.681	0.118
PIV	0.615 (0.552–0.678)	210.291	0.567	0.656	0.522	0.696	0.223
TPSA	0.661 (0.599–0.724)	11.577	0.480	0.807	0.622	0.701	0.288
Model A	0.750 (0.696–0.804)	0.362	0.772	0.625	0.576	0.805	0.397
Model B	0.742 (0.685–0.798)	0.399	0.724	0.693	0.609	0.792	0.417
Model C	0.751 (0.696–0.806)	0.428	0.669	0.750	0.639	0.774	0.419

MLR: monocyte-to-lymphocyte ratio; SII: systemic immune-inflammation index; PIV: pan-immune-inflammation value; AUC: the area under curve; ROC: receiver operating characteristic; PCa: prostate cancer; CSPCa: clinically significant prostate cancer, which was defined as Gleason grade ≥ 2; PPV: positive predictive value; NPV: negative predictive value; Model A: multivariable logistic regression analysis based on the MLR; Model B: multivariable logistic regression analysis based on the SII; Model C: multivariable logistic regression analysis based on the PIV.

## Data Availability

The original contributions presented in the study are included in the article/Supplementary Material. Further inquiries can be directed to the corresponding author.

## Supplementary Materials

The following are available online at https://www.mdpi.com/article/10.3390/jcm12030820/s1, Figure S1: Calibration curves of the nomogram for PCa detection in the training, and validation cohorts, Figure S2: Decision curve analysis of the nomogram for PCa detection.
