# Supplementary material for: Diagnostic Efficiency of Pan-Immune-Inflammation Value to Predict Prostate Cancer in Patients with Prostate-Specific Antigen between 4 and 20 ng/mL
Source: J Clin Med. 2023 Jan 19;12(3):820. doi: 10.3390/jcm12030820 (PMC9917630; doi:10.3390/jcm12030820)
Supplement: Supplementary file 1 [file jcm-12-00820-s001.zip › jcm-2101172-supplementary.pdf]

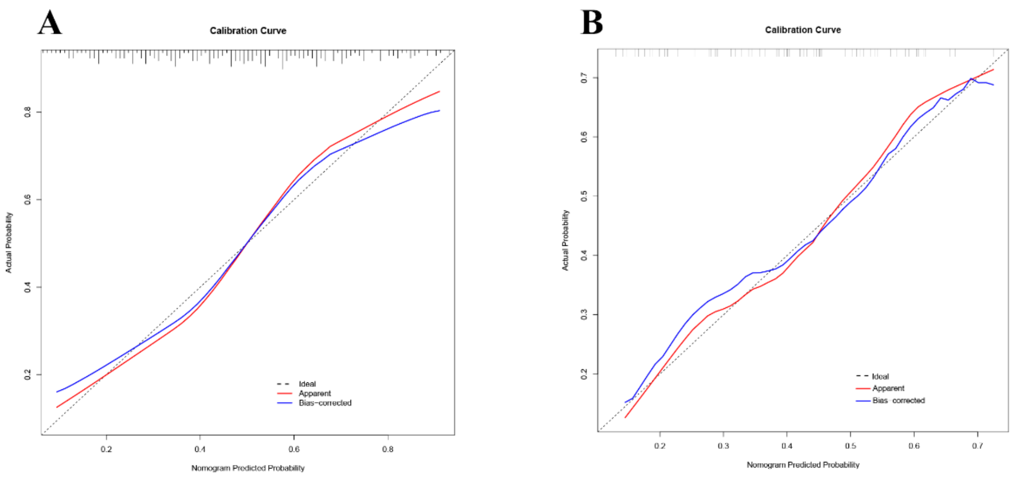

Supplementary Figure S1: Calibration curves of the nomogram for PCa detection in the training, and validation cohorts. A. Calibration curves of the nomogram in the training cohort. B. Calibration curves of the nomogram in the validation cohort.

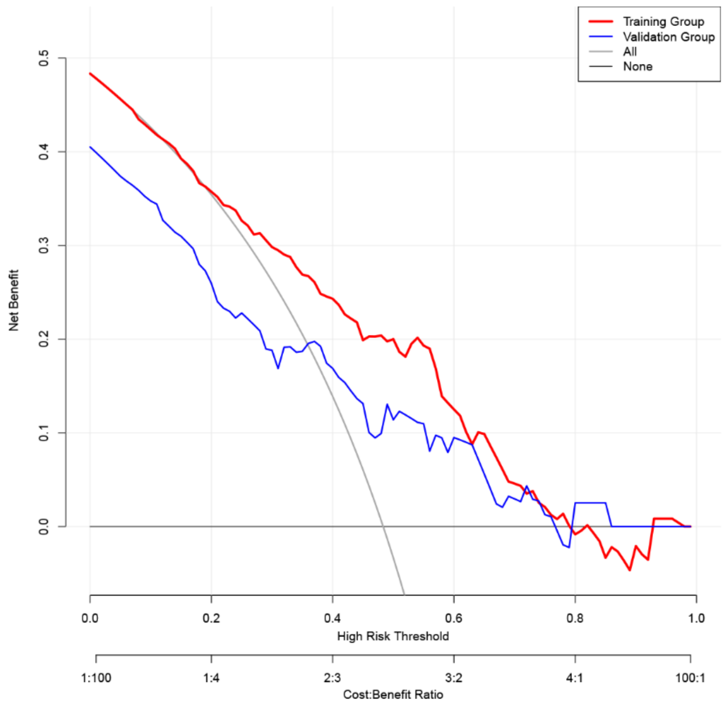

Supplementary Figure S2: Decision curve analysis of the nomogram for PCa detection.
